# Gut microbiota has the potential to improve health of menopausal women by regulating estrogen

**DOI:** 10.3389/fendo.2025.1562332

**Published:** 2025-06-09

**Authors:** Haiqiang Wang, Fan Shi, Lihong Zheng, Wenhui Zhou, Bowen Mi, Siyu Wu, Xiaoling Feng

**Affiliations:** ^1^ Department of Internal Medicine, First Affiliated Hospital, Heilongjiang University of Chinese Medicine, Harbin, China; ^2^ Graduate School of Heilongjiang University of Chinese Medicine, Harbin, China; ^3^ Department of Internal Medicine, Fourth Affiliated Hospital, Heilongjiang University of Chinese Medicine, Harbin, China; ^4^ Department of Gynecology, The First Affiliated Hospital of Heilongjiang University of Chinese Medicine, Harbin, China

**Keywords:** gut microbiota, menopause, estrogen, lipid metabolism disorder, cognition impairment, clinical application

## Abstract

Menopause is an age-related loss of ovarian function. As a woman enters menopause, the estrogen produced by her ovaries decreases, which will adversely affect women’s health. The symptoms related to menopause are related to the imbalance of gut microbiota. Studies have shown that the diversity of gut microbiota after menopause is lower than that before menopause, and the weakening of microbial decomposition will lead to the decrease of circulating estrogen, gradually resulting in disorders of lipid metabolism, cognitive decline, osteoporosis and other diseases. Gut microbiota play a key role in regulating estrogen levels. By secreting β-glucuronidase, it increases the reabsorption of estrogen in the enterohepatic circulation and mediates phytoestrogen metabolism, regulates estrogen homeostasis in the host and affects disease development and prognosis. Therefore, the gut microbiota is an overall regulator of women’s estrogen status during menopause and an untapped new area for improving women’s postmenopausal health. Changing the gut microbiota through specific prebiotics, probiotics, etc., and then affecting estrogen levels provides exciting opportunities for future therapeutic applications.

## 1 Introduction

Menopause is a special stage in a woman’s life when she transitions from childbearing to old age (1). During this period, as ovarian function gradually declines, estrogen levels fluctuate and eventually decline, leading to a series of physiological and psychological changes (2), and some symptoms that affect the quality of daily life (3). More seriously, menopause is also a risk factor for a number of diseases, such as metabolic diseases, cardiovascular diseases, osteoporosis, anxiety, depression, dementia and even cancer, which begin to appear and develop during menopause (4, 5). With the large number of menopausal women around the world, improving the quality of life of menopausal women and the prevention and treatment of menopause-related symptoms requires more attention. Hormone therapy (HT) has been widely used in the past and is an effective method to relieve menopausal symptoms (6). However, the clinical use of HT has become controversial with some experimental studies showing an increase in cardiovascular risk with combined estrogen-progestin use (7). Therefore, the choice of clinical treatment must be individualized, assessing patient risks and benefits (8). In addition to hormone replacement therapy, there are also non-hormonal drugs that can be used to relieve menopausal symptoms (9). More clinical research is needed to develop other new therapies to minimize future health risks for menopausal women.

The gut microbiota is closely related to women’s health. Together with the host, the gut microbiota forms a large, complex and dynamically changing micro-ecosystem that influences many physiological functions of the host by regulating the host’s immune response, maintaining the intestinal barrier function and resisting the invasion of pathogens (10–12). The gut microbiota is also affected by menopause. A meta-analysis systematically identified differences in the gut flora of premenopausal and postmenopausal women (13). A metagenome-wide association study showed that *Firmicutes* and *Roseburia* spp. are depleted, while *Bacteroidetes* and the toluene-producing genus *Tolumonas* are overrepresented in fecal samples from postmenopausal women (14). During the perimenopausal period, the relative abundance of beneficial bacteria such as *Lactobacillus* and *Bifidobacteria* is markedly reduced while that of harmful bacteria such as *Enterobacter* is increased in women (15). A large-scale survey of menopause and the gut microbiome suggests that the diversity of the gut microbiome is lower after menopause (16).

The effects of menopause on the gut microbiota are associated with a decrease in estrogen, and there is growing evidence that the gut microbiota and estrogen are bi-directionally regulated, with the gut microbiota being influenced by estrogen, and in turn, the gut microbiota significantly influencing estrogen levels (17). Studies have shown that estrogen supplementation during menopause slows the progression of atherosclerosis and corrects lipid metabolism disorders by regulating the abundance of gut microbiota (18). The gut microbiota regulates estrogen by secreting β-glucuronidase and participates in the metabolism of estrogen in the blood (14). Therefore, gut microbiota may be a therapeutic target for reducing risk in menopausal women.

The aim of this article is to elucidate the interactions that exist between menopause-estrogen-gut microbiota, to recognize the potential benefits of gut microbiota on menopause, and to identify and develop modulators targeting the gut microbiota to modulate estrogen, which may be useful in the treatment and prevention of menopause-related diseases and have a significant role in alleviating symptoms, improving quality of life and reducing mortality in menopausal women.

## 2 Decreased estrogen levels cause symptoms associated with menopause

**Figure 1:**
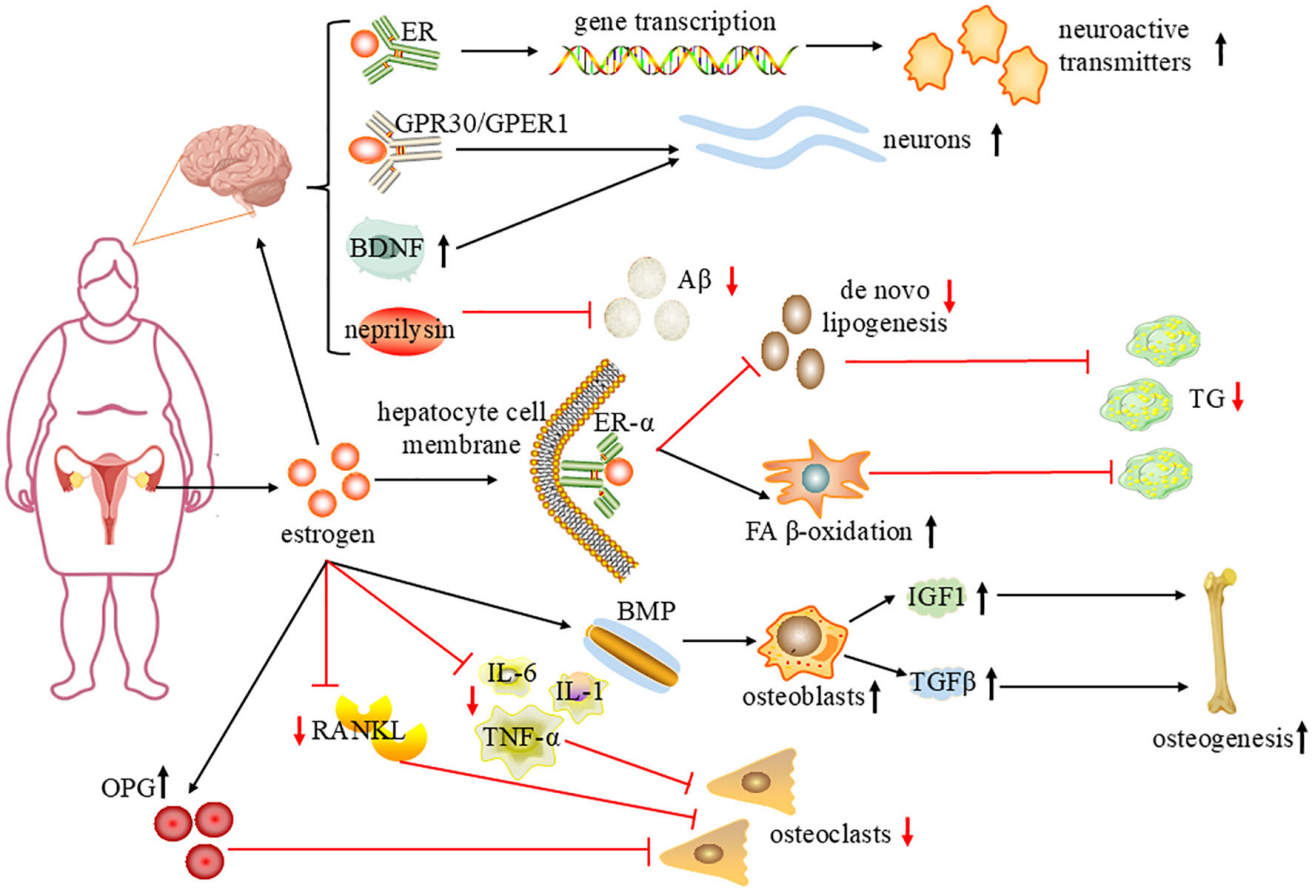
The mechanism of estrogen improving brain function, regulating lipid metabolism and alleviating osteoporosis. Estrogen is mainly secreted by the ovaries, In hepatocytes, estrogen binds to ERα, increasing FAβ-oxidation in mitochondria, reducing *de novo* lipogenesis, and reducing liver TG accumulation. Estrogen regulates gene transcription by interacting with nuclear receptors; activates different intracellular signal cascades through GPR30/GPER1 and regulates BDNF expression, protecting neurons; and regulates Neprilysin to degrade beta amyloid peptide (Aβ) and improve cognitive performance. Estrogen upregulates the bone morphogenetic protein (BMP) signaling pathway, stimulates osteoblasts to produce insulin-like growth factor I (IGF1) and transforming growth factor-β(TGFβ), promotes bone formation and remodeling; inhibits the expression of RANKL and IL-1, IL-6, TNF-α, promotes osteoprotegerin (OPG), thereby inhibiting the formation of osteoclasts. sharp arrows(→),stimulate; blunt arrows(⊥),inhibit; ↑, increase; ↓, decrease.

For menopausal women, many symptoms and signs related to menopause are caused by a lack of estrogen production, which brings a series of physiological and psychological challenges. Estrogen is an important sex hormone, mainly synthesized from cholesterol as the matrix, approximately 90% secreted by the ovaries, and a small amount produced by the adrenal gland and adipose tissue. 17-βestradiol (E2) is the main estrogen in the body and the most biologically active. Estrogen mainly exerts related biological effects by binding to estrogen receptor (ER) and plays a key role in regulating many physiological processes in the human body (**Figure 1**) (19). During a woman’s reproductive years, the average level of total estrogen is 100–250 pg/mL, however, circulating concentrations of E2 decline to 10 pg/mL after menopause (20). Therefore, estrogen reduction and gut microbiota imbalance interact, leading to the occurrence and progression of many diseases during menopause.

### 2.1 Lipid metabolism disorder

Lipid metabolism disorder refers to the abnormal increase or decrease of plasma lipids caused by various reasons and is a common pathological manifestation of menopausal women (21). It may induce stroke, coronary heart disease and other diseases, and is an important risk factor for cardiovascular disease, which seriously affects the health of menopausal women (22).

Ovariectomy was found to lead to elevated serum Low density lipoprotein cholesterol (LDL-C), excessive storage of glycogen and lipids in hepatocytes and alteration of the gut microbiota in female mice, demonstrating the important role of estrogens in the maintenance of glycolipid metabolism homeostasis (23). The liver is a central organ in the regulation of lipid and glucose metabolism, and ERα is the major receptor subtype in the liver. In hepatocytes, estrogen binds to ERα, increasing FA β-oxidation in the mitochondria and decreasing *de novo* lipogenesis, thereby reducing hepatic triglyceride(TG) accumulation (24). Estrogen has an important protective effect on adipose tissue distribution and fat metabolism. The decrease of estrogen level in women during menopause causes changes in the expression of key enzymes and genes in the process of lipid synthesis and decomposition, resulting in the increase of TG and LDL-C in serum and the decrease of high-density lipoprotein cholesterol (25, 26). In addition, it can also cause fat redistribution, which in turn leads to the rapid accumulation of visceral fat, forming central obesity (27). With the increase of visceral fat content, free fatty acids will also increase due to the excessive breakdown of fat, which leads to insulin resistance, triggering metabolic diseases (28). It has been demonstrated that estrogen deficiency after ovariectomy leads to reduced lipolysis and increased non-esterified fatty acid in white adipose tissue and brown adipose tissue, resulting in reduced fatty acid oxidation in the liver of ovariectomized(OVX) rats, which in turn leads to hepatic steatosis (29). The results of a cell-based study suggest that E2 directly activates SREBP2 gene expression, leading to excessive cholesterol accumulation and increased risk of cardiovascular disease (30).

Therefore, menopause is therefore a critical period during which alterations in lipid metabolism should be understood in order to reduce the risk of associated diseases caused by dyslipidemia.

### 2.2 Cognition impairment

Cognition impairment increases as menopause progresses, and the vast majority of women with dementia are postmenopausal (31). A study has confirmed that menopause impairs cerebrovascular function, which in turn leads to more widespread cognitive impairment in a mouse model of vascular contributions to cognitive impairment and dementia (32). Results of a cross-sectional study of older women in rural northern China show that earlier menopause is associated with poor cognitive performance and significantly increases the risk of mild cognitive impairment and dementia, particularly Alzheimer’s disease (AD), dementia with Lewy bodies and vascular dementia (33).

Cognitive decline is a consequence of reduced estrogen (34). J. Hu et al. found that higher blood levels of E2 were associated with lower rates of cognitive impairment in southeastern China (35). Other studies found that E2 levels and the expression of ERα, ERβ and GPER in OVX mice were significantly reduced, and there was a significant correlation with dyslipidemia and cognitive impairment, E2 supplementation or lipid lowering is an effective method to ameliorate postmenopausal hyperlipidemia induced hippocampal damage and cognitive impairment by upregulating ERs (36). Song X et al. study finds earlier use of oral contraceptives and hormone replacement therapy at menopause is associated with reduced risk of cognitive impairment (37).

Estrogen has neuroprotective effects, ranging from classical nuclear to non-classical membrane-mediated actions, and it affects the brain through complex cellular mechanisms. In the classic mechanism, estrogen regulates gene transcription by interacting with nuclear receptors, regulating the synthesis, release and metabolism of many neuroactive transmitters and the expression of their receptors. The nonclassical estrogen action is probably mediated by receptors integrated or associated with the cell membrane and by the activation of distinct intracellular signaling cascades through the high-affinity membrane-associated G protein-coupled estrogen receptor GPR30/GPER1, protecting neurons from excitotoxins and free radicals (38, 39). Estrogen can improve mitochondrial function (40). It has been shown that chronic ovariectomy reduces oxygen consumption, ATP production rates and mitochondrial membrane potential in NADH-associated respiration in Wistar adult female rats’hippocampal mitochondria (41). High levels of β-amyloid peptide (Aβ) in brain tissue are a risk factor for AD.17β-estradiol promotes Aβ degradation by regulating the expression of neprilysin, which may be a key factor in improving cognitive performance in menopausal women (42). Estrogen improves cerebral blood flow and provides beneficial clinical effects in cognitive functioning (43).

Overall, estrogen has been a major focus in the field of hormonal cognition as it prevents cognitive impairment by modulating neurotransmitters, protecting mitochondrial function, reducing Aβ formation, and increasing cerebral blood flow.

### 2.3 Emotional disorder

More and more research shows that menopausal women can be associated with emotional problems, such as anxiety and depression (44). A cross-sectional assessment of depression and anxiety in perimenopausal and menopausal women showed that 21.9% of women had moderate anxiety and 24.76% were clinically depressed (45). Women with emotion regulation disorders such as anxiety, depression and stress are more likely to develop severe menopausal symptoms that affect quality of life (46).

Estrogen has a significant effect on emotional disorders (47). Estrogen has the ability to modulate the release of neurotransmitters such as 5-hydroxytryptamine, noradrenaline, and dopamine, which is a key mechanism for antidepressant effects (48). brain-derived neurotrophic factor (BDNF) is a neurotrophic factor that is essential for maintaining brain function, there is a strong association between reduced levels of BDNF and the development of depression, and estrogen regulates BDNF expression, which promotes neurogenesis, synaptic plasticity, and neuronal survival and contributes to emotion improvement (49). Estrogen can also affect depressive behavior by regulating the disturbance of gut microbiota. *Enterococcus* in the stool of patients with Major depressive disorder (MDD) were significantly increased compared with that of healthy control group, which is related to pro-inflammation (50). Pathophysiological processes such as inflammation and immune activation, oxidative stress, and neurotransmitter synthesis in emotional disorders can be altered by regulating the gut microbiota (51). In addition, immune imbalance caused by declining estrogen levels during menopause is a new focus of research on menopausal depression, estrogen deficiency disrupts immune homeostasis through the ERα/ERβ/Gper-associated NLRP3/NF-κ B signaling pathway, leading to elevated levels of inflammatory cytokines, resulting in disruption of the blood-brain barrier, neurotransmitter dysfunction, impaired BDNF synthesis, and reduced neuroplasticity (52). Estrogen inhibits the production and release of inflammatory molecules in the body and protects nerve cell integrity by reducing inflammatory responses (47).

Estrogen regulates emotion by promoting the release of neurotransmitters, upregulating BDNF expression, maintaining the balance of the gut microbiota, and suppressing inflammatory factors. However, more clinical research is needed to clarify the best way to use estrogen therapy to relieve menopausal emotional disorders.

### 2.4 Osteoporosis

Recent research has increasingly emphasized the importance of the gut microbiota in maintaining bone homeostasis (53). A study on the gut microbiota of postmenopausal women with osteoporosis in Shanghai, China, showed that the content of four bacteria genera, *Roseburia*, *Clostridia_UCG.014*, *Agathsium* and *Dialister*, were lower in the osteoporosis group than in the normal control group. They are all microorganisms with potential anti-osteoporosis properties (54). Osteoporosis (OP) is a systemic bone disease characterized by low bone mineral density (BMD) and deterioration of bone structure, leading to reduced bone strength and thus increased susceptibility to fracture (55). OP is more common in postmenopausal women, and menopausal estrogen deficiency is a major risk factor for postmenopausal osteoporosis (56). The cellular component of bone includes osteocytes, osteoblasts, and osteoclasts, each playing essential roles in bone integrity and remodeling (57). Decreased estrogen results in increased osteoclast differentiation and activation, accelerated bone resorption over the rate of formation and rapid bone loss, making bone brittle and prone to fracture (58).

Estrogen regulates the bone morphogenetic protein (BMP) signaling pathway, which is crucial for osteoblast differentiation and bone formation. In addition, estrogen stimulates osteoblasts to produce insulin-like growth factor I (IGF1) and transforming growth factor-β (TGF-β), further promoting bone formation and remodeling (59). Estrogen inhibits the expression of the receptor activator of NF-kB ligand (RANKL), a key factor in the activation of osteoclasts, and promotes osteoprotegerin (OPG) production. OPG acts as a decoy receptor for RANKL and directly inhibits osteoclast formation (60). In addition, estrogen inhibits cytokines such as interleukin-1(IL-1), interleukin-6(IL-6), and tumor necrosis factor-α(TNF-α), which indirectly inhibits osteoclast differentiation (61). Estrogen deficiency increases apoptosis of osteoblasts and also alters the mechanical responsiveness of MLO-Y4 osteoblasts, ultimately reducing osteoblast differentiation and function (62).

Overall, estrogen is a key factor in maintaining the health of menopausal women, and estrogen deficiency can lead to the development of a variety of menopause-related diseases. How to regulate hormonal changes during menopause and provide the body with the necessary hormones is a major area of research to alleviate menopausal diseases. Recent research suggests that the gut microbiota may play a key role in this.

## 3 Gut microbe-estrogen axis: gut microbiota regulates estrogen levels

The relationship between estrogen and gut microbes is an expanding area of research that may lead to new therapeutic options for a variety of conditions associated with estrogen deficiency during menopause. A growing body of research suggests that the relationship between estrogen and the gut microbiota is bidirectional (63).

The intestinal protective effect of estrogen reduces intestinal permeability by up-regulating the expression of tight junction proteins (64). Inhibit the NF-κ B pathway, reduce pro-inflammatory cytokines, reduce intestinal inflammation, promote the abundance of beneficial bacteria, and maintain intestinal homeostasis (65). Estrogen levels can affect the diversity of gut microbiota, it has been shown that the lack of ER β induces a dysregulation of the intestinal ecology, leading to anxiety and depression-like behaviors in mice (66). The abundance of *Aggregatibacter segnis*, *Bifidobacterium animalis* and *Acinetobacter guillouiae*, which are associated with sex hormone levels, were all found to be decreased in the gut microbes of patients with menopausal syndrome (67). The experimental results showed that supplementing a small amount Low dose brain estrogen can maintain the diversity of intestinal microorganisms in estrogen-deficient rats (68).

**Figure 2:**
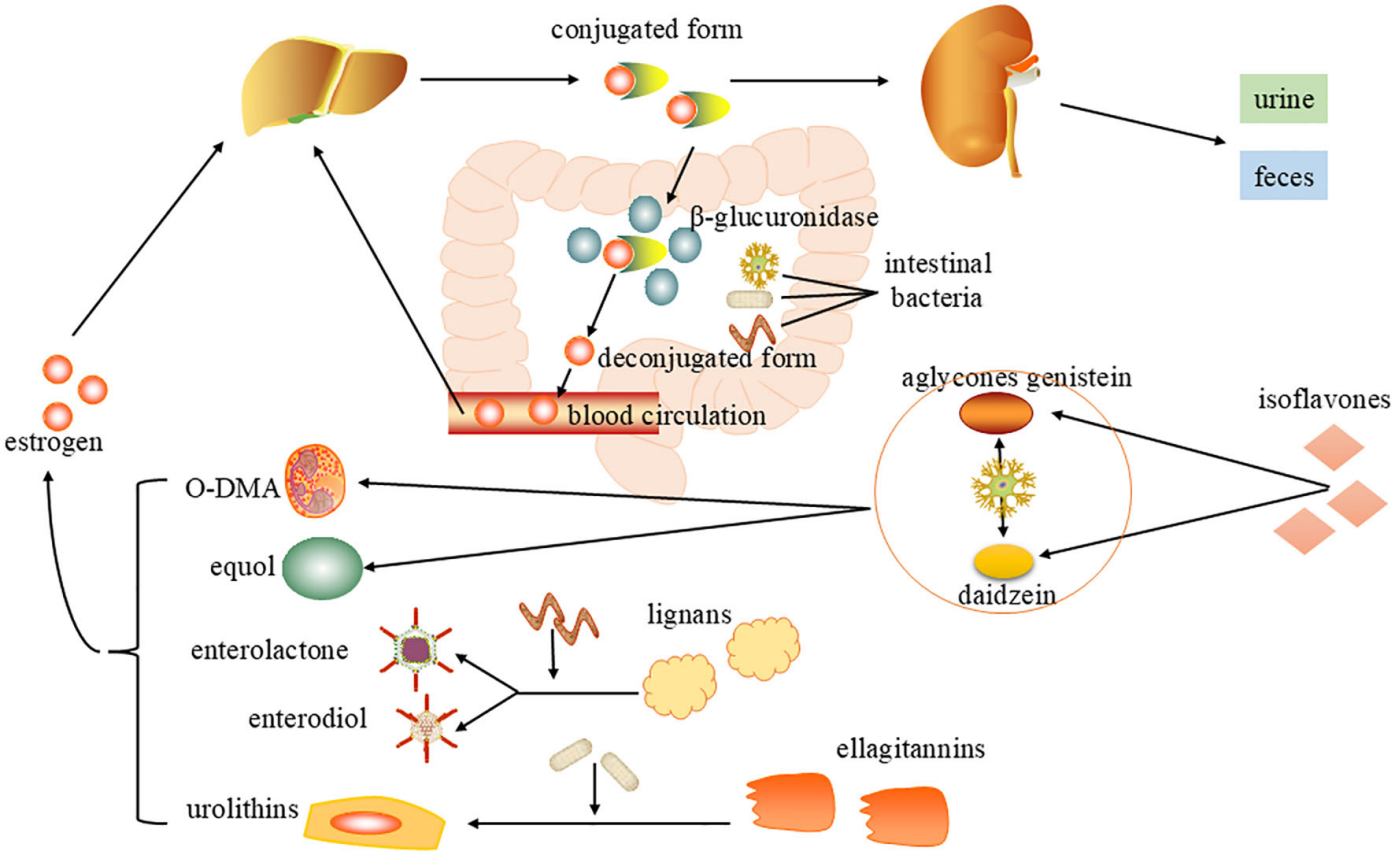
Mechanisms of gut microbiota regulation of estrogen levels. After metabolism by the liver, estrogen enters the intestine in bile as an inactive conjugated form and is metabolized by β-glucuronidase (gmGUS) secreted by intestinal microorganisms to an active deconjugated form, which is reabsorbed through the intestinal mucosa into the blood circulation and returned to the liver. Gut microbes can also convert phytoestrogens into estrogen analogues. Through bacterial metabolism, isoflavones can be transformed into equol and O-desmethylangolensin (O-DMA). Lignans are converted into enterolactone(ENL) and enterdiol(END). Ellagitannins transform a series of compounds called urolithins, and the bioavailability and biological activity of these microbial-derived compounds have both been enhanced.

Gut microbiota participate in the estrogen metabolic cycle through β-glucuronidase and can also convert phytoestrogens into estrogen analogues to regulate estrogen levels (**Figure 2**). See the following text for details.

### 3.1 Gut microbiota increases estrogen reabsorption in enterohepatic circulation

Circulating estrogens are highly regulated by symbiotic bacterial activity, the human gut microbiota regulates estrogen metabolism through the “estrobolome” that is a collection of bacterial genes that encode enzymes like β-glucuronidases and β-glucosidases. These enzymes increase the reabsorption of active free estrogens into the bloodstream in the enterohepatic circulation, affecting circulating levels and are important mediators of gut microbiota-host interactions (69). Estrogens are metabolized mainly in the liver, forming biologically inactive conjugated form that are excreted in the bile and eventually enter the intestine, where they are partly excreted in the feces and urine. The enzyme β-glucuronidase (gm GUS) secreted by gut microbiota metabolizes estrogen from its conjugated form to its unconjugated form, restores its activity, and is reabsorbed through the intestinal mucosa into the blood circulation and back to the liver, a process known as the enterohepatic circulation of estrogen (70). Both the increased number of bacteria with “estrobolome” and the increased activity of these gene-encoding enzymes can accelerate the early dissociation and hydroxylation of estrogens in the intestine so that the free estrogens could increase significantly in enterohepatic circulation and maintain at a physiological level (71). Conversely, if the gut microbiota is imbalanced and microbial diversity is reduced, β-glucuronidase activity is reduced and the enterohepatic circulation is compromised, leading to a reduction in circulating estrogens (72). A randomized controlled trial demonstrated that supplementation with a probiotic formula with β-glucuronidase activity regulated serum estrogen levels in healthy postmenopausal women compared to a placebo group, setting the stage for future use of probiotics in the postmenopausal population (73).

### 3.2 Conversion of phytoestrogens to estrogen analogues by gut microbes

Phytoestrogens are a class of plant produced polyphenolic compounds with diphenolic structure. Its structure is similar to that of the major estrogen 17-βestradiol, which binds to the estrogen receptor and has estrogen agonist and estrogen antagonist effects (74). When estrogen levels decrease, these plants increase, offering a gentle boost to maintain hormonal harmony. In situations with high estrogen concentrations, phytoestrogens work by blocking stronger estrogens from binding to receptors and keeping things in check. Common sources of phytoestrogens include soybeans, flaxseed, grains, fruits, and vegetables, which are synthesized through the phenylpropanoid pathway and subsequently transformed into diverse chemical structures through specific enzymes (75). Phytoestrogens are classified into seven groups: Isoflavones, flavones, flavanones, chalcones, coumestanes, lignanes and stilbenes. Among them, isoflavones, lignans, and coumestans are the main bioactive types (76).

Phytoestrogens have been shown to have a variety of health benefits for humans such as antioxidants, neuroprotection, immune system enhancement, cardiovascular protection and more (77). A study of older adults in southern Italy found that higher intake of phytoestrogens, particularly isoflavones, was associated with better cognitive performance (78). Phytoestrogens can also improve bone density, alleviate menopausal vascular relaxation symptoms, and have a certain therapeutic effect on menopausal related diseases (79). Despite the various health benefits, the bioavailability of phytoestrogens in the human body is low, as most of the ingested phytoestrogens are not absorbed in the small intestine, and the effects of phytoestrogens on organisms are largely mediated by the gut microbiota, which are metabolized by gut bacteria to modulate phytoestrogen activity and increase their bioavailability (80).

Plant isoflavones are the most famous of all phytoestrogens and have attracted attention due to their health properties, isoflavones are transformed in first place into the aglycones genistein and daidzein. Daidzein can be metabolized by a few bacteria like *Adlercreutzia equolifaciens*, *Eggerthella* sp. *YY7918*, *Lactococcus garvieae*, *Slackia equolifaciens*, *Slackia isoflavoniconvertens*, *Slackia* spp. into equol and O-desmethylangolensin (O-DMA) (81). Lignans are not easily absorbed by the intestine and must be metabolized into enterolactone(ENL) and enterdiol(END) by gut microbiota such as *Clostridiumsaccharogumia*, *Eggerthella lenta* and *Blautia producta*, which are called enterolignans, before they can enter the body and play their roles (82). Ellagitannins and its hydrolyzed product ellagic acid are difficult to be absorbed by the blood, gut bacteria gradually metabolize it by means of lactonering cleavage, decarboxylation and dehydroxylation reactions, which lead to the formation of a series of compounds named urolithins (83). These microbe-derived compounds have improved bioavailability and bioactivity with higher estrogenic/anti-estrogenic, antioxidant, anti-inflammatory and anti-tumor activities (84). However, it should be noted that phytoestrogens can also have some side effects, such as allergic reactions in some people, so moderate intake is crucial (85).

The interaction between gut microbiota and phytoestrogens is bidirectional. Phytoestrogens are metabolized by the gut microbiota on the one hand, and on the other hand, the metabolites formed regulate and reshape the gut microbial composition by altering the diversity and abundance of bacteria (86). In summary, the gut microbiota plays a very important role in determining the absorption, metabolism, distribution and excretion of ingested phytoestrogens and their metabolites (87).

There is growing evidence that the gut microbiota plays an important role in estrogen regulation, which may be an untapped new area of women’s health before and after menopause (73). Therefore, a better understanding of the changes in the gut microbiota during menopause and the development of specific probiotic strains to modulate the gut microbiota, which in turn affects estrogen levels, could be a potential therapeutic approach to counteracting the changes in estrogen during the menopausal transition and improving women’s health.

## 4 Clinical implications of gut microbiota modulation in menopause-associated disorders

The gut microbiota consists of trillions of complex and dynamic microorganisms, and the pathogenesis of many human diseases may be related to the ‘dysbiosis’ of the gut microbiota, which is manifested by a decrease in beneficial bacteria, an increase in harmful bacteria, and a loss of compositional and functional diversity (88). Therefore, gut microbes have been considered as possible therapeutic targets to address menopause-related diseases. Probiotic supplementation may be a viable and safe strategy for the treatment of menopause-related disorders. In particular, oral probiotic formulations—especially those including *Lactobacillus ssp. casei*, *helveticus*, *rhamnosus* and *reuteri*, may have multiple beneficial effects on health (89). Besides, dietary interventions, fecal microbiota transplantation (FMT), exercise or drugs, have been progressively applied in clinical or preclinical studies (90).

### 4.1 Probiotic

#### 4.1.1 Improve estrogen activity and adjust lipid metabolism disorder

Probiotics multiply the gut and produce beneficial gut bacterial metabolites such as short-chain fatty acids (SCFAs), restoring the normal functional activity of the gut microbiota, improving glycolipid metabolism and increasing serum 17β-estradiol concentrations (89). 17β-estradiol can be activated by PI3K/AKT signaling mediated by ERβ and GPR30 to alleviate postmenopausal dyslipidemia (91), and also upregulates the ERα/SIRT1/PGC-1α signaling pathway, protecting mitochondrial function and preventing lipoatrophy (92). In one study, supplementation with the probiotic *B. longum 15M1*, as well as a combination of *Lactobacillus plantarum 30M5* and a diet of *soy isoflavones* (SIFs), both alleviated lipid metabolism disorders during menopause (93). It has been demonstrated that butyric acid supplementation restores PPARα activity in high fat diet fed rats, which enhances fatty acid β-oxidation, inhibits lipid synthesis, and down-regulates nuclear factor κB pathways and inflammation, alleviating hepatic steatosis (94).

#### 4.1.2 Regulating neurotransmitters to alleviate emotional disorders

The study showed that the degradation of estradiol by gut microbes containing 3β-hydroxysteroid leads to a decrease in serum estradiol levels, which leads to depression in female mice, revealing that gut microbes may be a new intervention target for depression (95). *Bifidobacterium* and *Lactobacillus* have been shown to improve emotion disorders such as anxiety, depression and stress in animal studies (96). Increased levels of glutamate and N-acetyl aspartate in the brains of mice treated with *L. rhamnosus JB-1* provide direct evidence that probiotic treatment modulates neurotransmitter concentrations to influence brain activity (97). Oral administration of a probiotic formulation, consisting of *L. helveticus R0052* and *B. longum R0175*, over 30 days was shown to improve mood in generally healthy volunteers (96). *Lactobacillus Calmette-Guerin (CP2305)* is a paraprobiotic that has been shown to improve psychological symptoms specific to menopausal women in a controlled clinical trial (98).

#### 4.1.3 Increase beneficial gut bacteria and inhibit bone loss

The regulation of bone metabolism by probiotics and prebiotics is gradually becoming a hot research topic. *Prevotella histicola* prevents estrogen deficiency-induced bone loss via the gut-bone axis in postmenopausal women and OVX mice, promising as a therapeutic target for osteoporosis treatment (99).Prebiotics are the food components that are fermented by gut microbiota, resistant to gastric acid and hydrolytic enzymes, but not digested and absorbed by the intestine, and prebiotics can also selectively modulate the activity of one or more beneficial gut microbiota to hos (100).Inulin-type prebiotics can increase the number of beneficial bacteria in the intestine and promote the release of organic acids, thus reducing the pH value of intestine, promoting the absorption of minerals, and inhibiting the bone loss (101).

### 4.2 Herbal extracts

Some herbal extracts can also act on the gut microbiota, further exerting pharmacological effects. Radix angelica dahuricae (RAD) is a well-known traditional Chinese medicine that can attenuate estrogen deficiency-induced dyslipidemia, improve TC and TG levels, and reduce hepatic TNF-α, IL-6 and IL-1β gene expression in OVX rats by modulating the composition of the gut microbiota and bile acid signal (102). Trifolium pratense ethanolic extract (TPEE) improves gut microbiota composition in OVX rats, mainly including *Firmicutes* and *Bacteroidetes*. The abundance of *Bacteroidetes* in the gut helps maintain healthy blood cholesterol levels. TPEE also increases the abundance of *Lactococcus* sp. and converts biochanin A and formononetin into equol, thereby enhancing estrogen activity. TPEE-treated OVX rats showed significant reduction in TG and LDL levels (103).

However, it should be noted that the effectiveness of these extracts is mostly reflected in animal experiments, the lack of human studies to further verify the differences between animals and humans in the physiological structure and metabolic pathways, etc. In the future, more clinical trials are needed to validate the effectiveness and safety of these extracts on the human body before they can be further applied to clinical treatment.

### 4.3 Adjust exercise and diet patterns

Exercise has the ability to alter the composition and function of the gut microbiota. Studies in animal models suggest that wheel running exercise training enhances gut microbial abundance and has multiple beneficial effects on the gut microbiota, and may be a promising strategy for the treatment of cognitive impairment in menopause (104).

Adjusting dietary patterns is a relatively effective and healthy option for intervening in the gut microbiota and has significant modulatory effects on bone metabolism (105).Higher dietary protein intake increases the abundance and diversity of the gut microbiota, whereas fat-rich diets may promote bile secretion, have a detrimental effect on bacterial cell membranes, and play a negative role in the metabolic regulation of osteoporosis (106). Therefore, dietary intervention may become a more economical, effective and simple way to reduce side effects in the future.

### 4.4 Other intervention measures

The phytoestrogen Secoisolariciresinol diglucoside (SDG) promotes the production of the gut microbial metabolites END and ENL, inhibits cerebral Aβ deposition, activates GPER to enhance the CREB/BDNF signaling pathway and suppresses the neuroinflammatory response to ameliorate cognitive deficits (107). Supplementation with dimethyl itaconate (DI) improved changes in the gut microbiota of mice fed a high-fat diet, increasing the abundance of bacteria that produce propionic acid and butyric acid, thereby improving cognitive function (108).

Fecal microbiota transplantation (FMT) has been used exploratively in osteoporosis control studies in recent years and is a promising therapeutic option for osteoporosis (109). The results showed that FMT lasting 8 weeks prevented OVX-induced bone loss by optimizing the composition and abundance of gut microbiota, increasing SCFA levels, and suppressing the release of pro-osteoclastogenic cytokines (110).

In summary, The role of the gut microbiota is not limited to the gut, but extends even to the liver through the gut-liver axis to regulate metabolism (111), to the central nervous system through the gut-brain axis (112), and to the bone metabolism and maintenance of bone health through the gut-bone axis (113). Therefore, interventions such as ingestion of probiotics and prebiotics, adjustment of diet and exercise patterns, and FMT can improve the composition and abundance of gut microbiota and related metabolites to varying degrees, which may provide new ideas for the prevention and treatment of menopausal diseases.

## 5 Conclusion

There is growing evidence that gut microbiota and estrogen have complex bidirectional effects that have significant implications for menopausal women’s health. The decrease of estrogen level in menopause will affect the composition and function of gut microbes, and lead to lipid metabolism disorders, cognitive disorders, emotional disorders and osteoporosis, which seriously affect the quality of life. Gut microbiota also has a regulatory effect on estrogen levels, and this paper takes the intervention of gut microbiota as an entry point, and summarizes a variety of methods that can regulate the gut microbiota in the clinic, including supplementation of probiotics and prebiotics, herbal extracts, dietary interventions, and FMT, which have demonstrated satisfactory results in alleviating symptoms associated with menopause. Research on the gut microbiota provides new insights and treatments to improve the health of menopausal women, and has the potential to bring new benefits to postmenopausal women’s health.
